# Hole, Convex, and Silver Nanoparticle Patterning on Polystyrene Nanosheets by Colloidal Photolithography at Air–Water Interfaces

**DOI:** 10.1021/acs.langmuir.2c01069

**Published:** 2022-06-22

**Authors:** Rino Kaneko, Hiroto Ichikawa, Marika Hosaka, Yoshihiro Sone, Yoshiro Imura, Ke-Hsuan Wang, Takeshi Kawai

**Affiliations:** Department of Industrial Chemistry, Tokyo University of Science, 1-3 Kagurazaka, Shinjuku-ku, Tokyo 162-8601, Japan

## Abstract

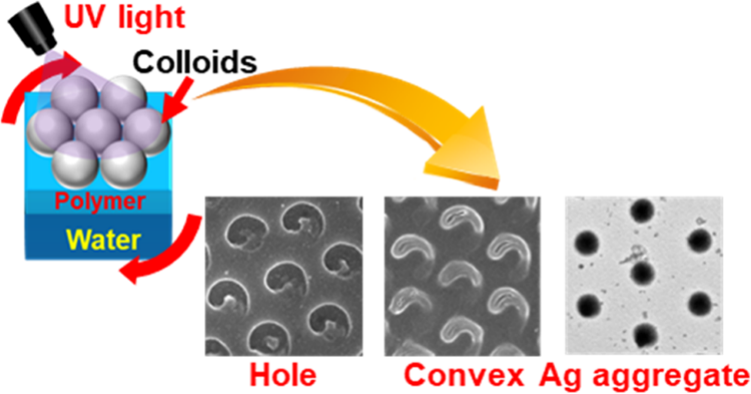

Colloidal photolithography is a versatile advanced technique for fabricating periodic nanopatterned arrays, with patterns carved exclusively on photoresist films deposited on solid substrates in a typical photolithographic process. In this study, we apply colloidal photolithography to polystyrene (PS) films half-covered with poly(methyl methacrylate) (PMMA) colloids at the air–water interface and demonstrate that periodic hole structures can be carved in PS films by two processes: photodecomposing PS films with ultraviolet (UV) light and removing PMMA colloids with a fluorinated solvent. Nonspherical holes, such as C-shaped and chiral comma-shaped holes, are also fabricated by regulating the UV illumination conditions. Furthermore, in addition to holes, convex patterns on PS films are realized by combining weak UV illumination with solvent treatment. We also demonstrate that actively using the water surface as the UV illumination field enables periodic silver nanoparticle spots to be deposited on PS films simply by dissolving silver ions in the water phase.

## 1 Introduction

Colloidal lithography is an advanced facile, inexpensive, and high-throughput technique for fabricating various periodic micro- and nanostructural arrays, including nanoholes, nanopillars, nanorings, and even asymmetrically shaped patterns.^1−9^ In this technique, single- or multilayered colloidal crystals are employed as templates or masks that are first deposited on a processable substrate, which is then subjected to the main nanofabrication process, such as deposition,^10−16^ reactive-ion etching (RIE),^17−21^ etchant-based wet etching,^22^ and illumination with ultraviolet (UV) light.^23−32^ Further postprocessing is generally required to construct complex target structures on the substrate, in which the processing steps depend on the target structure.

UV-light-assisted colloidal lithography, i.e., colloidal photolithography, is one of the most widely used colloidal lithography techniques because it can be used under atmospheric conditions without special processing equipment.^23−32^ In conventional colloidal photolithography, UV light is focused on small photoreactive layer spots underneath a colloidal monolayer, leading to the formation of patterns in the photoreactive film. Furthermore, oblique illumination with UV light shifts the focusing position from just under the colloid to one that is not in contact with the colloid,^26^ enabling patterns to be carved at desired positions on the underlying film. Positive or negative photoresists have been exclusively used as underlying films;^23−32^ consequently, adapting conventional polymers for use in colloidal photolithography would greatly expand its applicability.^33,34^

**Figure 1:**
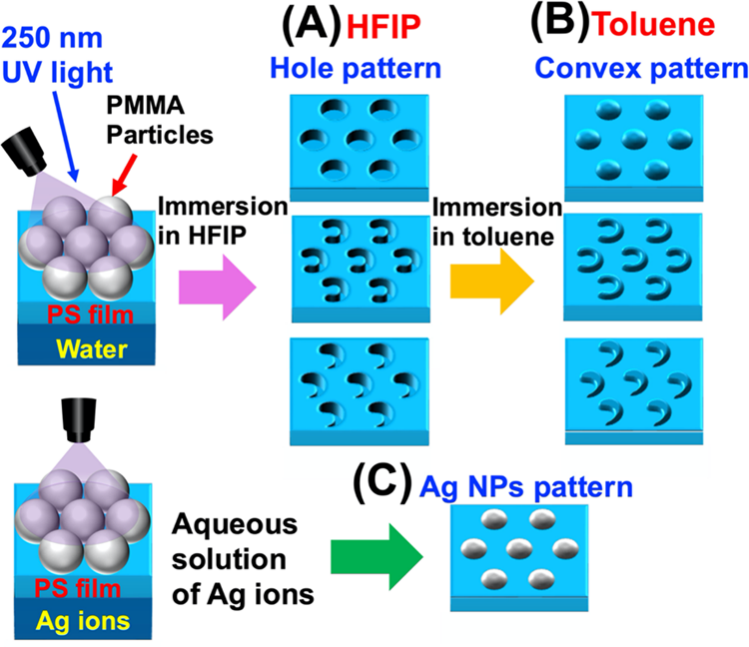
Schematic showing the preparation of various nanopatterns by colloidal photolithography at the air–water interface. Pattering of (A) holes by treatment with a fluorinated solvent (HFIP), (B) convex structures by treatment with HFIP and toluene, and (C) Ag NP aggregates formed using a water phase containing Ag ions.

Colloidal photolithography is expected to form nanopatterns when UV light corresponding to the absorption band of a polymer is used because the exposure to UV light can promote the efficient photo-oxidative degradation of the polymer through polymer-chain breakage and radical formation.^35,36^ In this study, we performed colloidal photolithography without any photoresist film using polystyrene (PS) films half-covered with poly(methyl methacrylate) colloidal particles (PMMA CPs), i.e., PS films covered with PMMA CPs on one side, at the air–water interface (Figure 1) and demonstrated that irradiation with 250 nm UV light, which corresponds to the absorption band of PS, effectively creates a periodic hole array in the PS film.^35,36^ We also report that judicious combinations of UV irradiation angle and PS-film rotation give rise to nonspherical holes (Figure 1A), C-shaped, or chiral comma-shaped holes that exhibit circular dichroism.^37−48^ Those shaped hole patterns have been fabricated using template-assisted lithography, focused electron beam, and hole-mask lithography;^37−48^ however, there are few reports of processing those patterns on polymeric materials. Such patterned polymeric materials may be readily employed in flexible devices due to their inherent flexibility. Furthermore, because nanopatterns are produced by leaving or removing UV-irradiated photoresist domains in conventional colloidal photolithography, embossing nanopatterns on photoresist films is impossible. We show that illumination with weak UV light and subsequent organic solvent treatments enables the production of periodic convex structures on PS films (Figure 1B).

The ability to transfer from one substrate to another is a highly desirable characteristic that can extend the versatility of a patterned film. However, for patterned films produced on solid substrates using conventional colloidal lithographic methods, transferring the patterned film to another substrate by peeling it from the original substrate is difficult due to strong adhesion. To overcome this problem, we used the water surface as an alternative substrate. Specifically, PS films at air–water interfaces were subjected to UV-irradiation-assisted patterning; the patterned PS films were found to stick to various substrates, including curved ones, without any peeling process, providing further postprocessing opportunities for the construction of more complex and elaborate nanopatterns.^49−51^ By focusing the UV light, photoreactive products can be deposited in small areas by simply solubilizing photoreactive compounds in the water phase, which is another advantage of a water surface as the processing substrate (Figure 1C). Herein, we report the use of an aqueous solution of silver ions (as the photoreactive compound) to fabricate periodic spot arrays of Ag nanoparticle aggregates on PS films.

## 2 Experimental Section

### 2.1 Materials

Polystyrene (average polymerization degree *n* = 1000–1400; Nacalai Tesque), Triton X-100 (Sigma–Aldrich), 1,1,1,3,3,3-hexafluoro-2-propanol (HFIP, Tokyo Chemical Industry), and CH_3_COOAg (Kanto Chemical) were used as received. Poly(methyl methacrylate) (PMMA) particles of 800 and 1500 nm in diameter were obtained from Soken Chemical & Engineering. Distilled water was used in all experiments.

### 2.2 Preparing Polystyrene (PS) Films Half-Covered with PMMA Colloidal Particles (CPs)

PS films (∼250 nm thick) on glass slides were prepared by spin-coating a toluene solution of 4 wt % polystyrene at 3000 rpm. PS film thickness was controlled by the concentration of the PS solution in toluene, with concentrations of 3, 4, 5, 7, and 9 wt % affording thicknesses of 100, 250, 300, 500, and 700 nm, respectively. Subsequently, PMMA CPs dispersed in 2:1 water/ethanol (prepared by sonicating a mixture of PMMA CP powder (1 g) in water (10 mL) for 10 min, after which the dispersion was mixed with 30 mL of Triton X-100 (0.25 wt %) in ethanol and sonicated for 5 min before use) were spin-coated at 1500 rpm onto each PS film to provide close-packed PMMA CPs deposited on the film. The resulting arrangement of PMMA particles is shown in Figure S1a,b.

### 2.3 Fabricating Holes and Ag Spots by UV Irradiation

PS films half-covered with PMMA CPs (PMMA CPs/PS films) were peeled off by diagonally sliding the glass-slide-deposited film into water. The peeling process was performed within 30 min after the PMMA CPs/PS film was fabricated on the glass substrate; otherwise, the film was difficult to peel off. The PMMA CPs/PS film was floated in a homemade Teflon container (30 × 30 × 15 mm^3^) filled with water. The floating films were exposed to UV light using a quartz optical fiber; the UV light was generated by passing light from a 200 W mercury xenon lamp (LA-410UV, Hayashi-Repic Co., Ltd.) through an optical bandpass filter. The UV-irradiated PS films on water were scooped onto glass slides or other substrates, after which they were immersed in HFIP for ∼5 min to remove the PMMA particles (Figure S1e).

Ag nanoparticles (Ag NPs) were deposited onto the PS films using a 1 mM CH_3_COOAg solution used instead of water. The UV irradiation process and the removal of PMMA particles by HFIP were the same as the method used to fabricate holes.

### 2.4 Fabricating C-Shaped and Comma-Shaped Hole Patterns

The Teflon container with the PMMA CPs/PS film floating on the water surface was placed in the center of a precision motorized rotation stage (PRMTZ8, Thorlabs). The stage was rotated at a given speed while the film was irradiated with UV light at an incident angle of 30°. The UV-irradiated PS films on water were scooped onto glass slides or other substrates, after which they were immersed in HFIP for ∼5 min to remove the PMMA particles.

### 2.5 Fabricating Embossed Structures

The preparation of the PMMA CPs/PS film on water and the UV irradiation process is the same method used for the preparation of holes although a weak UV light (10 mW/cm^2^) was used. After the UV-irradiated PS films on water were scooped onto glass slides, they were immersed in HFIP for 5 min to remove PMMA particles and subsequently immersed in toluene for 5 min.

### 2.6 Characterization

The UV-irradiated PS films on water were scooped onto various substrates, namely, a copper grid coated with an elastic carbon film for transmission electron microscopy (TEM), a CaF2 substrate for Fourier transform infrared (FTIR) spectroscopy, a quartz plate for ultraviolet–visible (UV–vis) and circular dichroism (CD) spectroscopies, and a Si wafer for atomic force microscopy (AFM). TEM and scanning electron microscopy (SEM) were performed using a JEOL JEM-2100 microscope operating at 200 kV and a Hitachi S4800 microscope operating at 20 kV, respectively. The films were coated with osmium for SEM using a Meiwafosis Neoc Pro instrument. STEM-EDS maps were acquired using a JEOL 2100 system equipped with an EDX spectrometer operating at 200 kV. UV–vis, FTIR, and CD spectra were acquired using a UV–vis spectrometer (JASCO, V-570), an FTIR spectrophotometer (Thermo Scientific, Nicolet 6700), and a circular dichroism spectropolarimeter (JASCO, J-820), respectively. AFM images of convex structures on PS films were obtained using an MFP-3D-BIOJ (Oxford Instruments Asylum Research) instrument operating in tapping mode. X-ray diffraction (XRD) patterns were recorded using a Rigaku Ultima IV diffractometer.

## 3 Results and Discussion

### 3.1 Hole-Patterned PS Films

**Figure 2:**
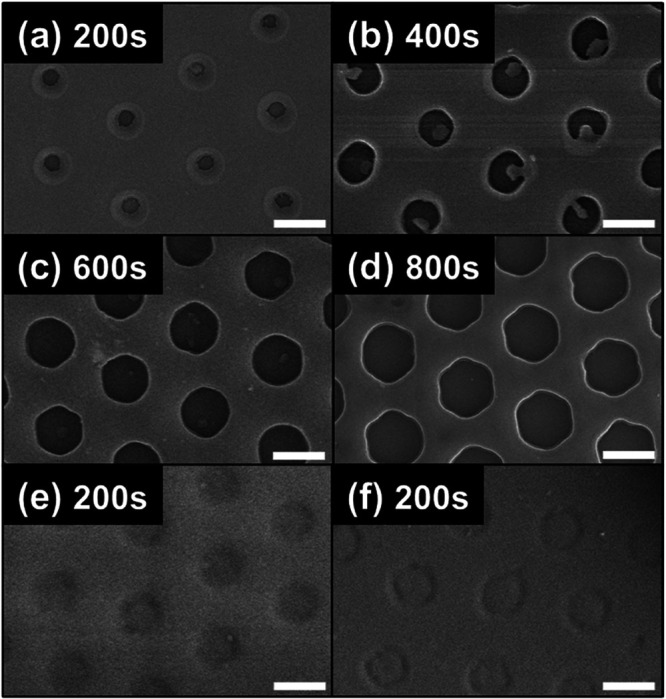
SEM images of UV-irradiated PS films after removing PMMA CPs with HFIP. The PMMA CPs are 1500 nm in diameter and the PS film is ∼250 nm thick. Irradiation times: (a) 200 s, (b) 400 s, (c) 600 s, and (d) 800 s at 30 mW/cm^2^ of 250 nm UV light. Irradiation times: 200 s at (e) 25 and (f) 15 mW/cm^2^. Scale bars: 1 μm.

PS films half-covered with PMMA CPs at the air–water interface were irradiated with 250 nm UV light, which corresponds to the absorption band of polystyrene (Figure 1). The UV-irradiated films processed on water were found to stick to various solid substrates, including filter paper, glass slides, and silicon wafers, simply by scooping each film with the desired substrate (Figure S2). Figure 2 shows SEM images of the PS films with PMMA CPs removed by washing with a fluorinated solvent (hexafluoroisopropanol, HFIP) for 5 min, highlighting the formation of periodic holes even in PS films. The holes gradually increased in size with increasing continuous exposure time using 30 mW/cm^2^ UV light (Figures 2a–d and S3), while a shorter exposure time with low-intensity UV light (25 and 15 mW/cm^2^) formed shallow hollows rather than holes (Figure 2e,f). Hole size was found to depend mainly on the total exposed UV energy rather than the intensity of the UV light (Figure S4). Here, similar hole pattens were formed when UV light was irradiated on PS films with PMMA CPs deposited on glass substrates instead of water surfaces, and subsequent HFIP treatment was performed (Figure S1c,d).

**Figure 3:**
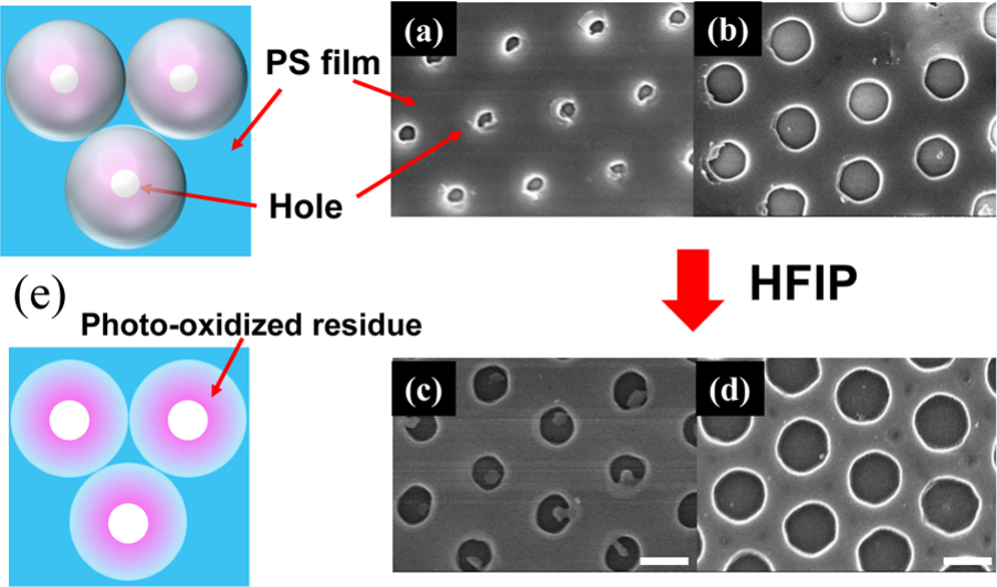
SEM images of UV-irradiated PS films (a, b) before and (c, d) after HFIP treatment, and (e) schematic illustrating the effect of HFIP treatment on hole size. UV light intensity: 30 mW/cm^2^. Irradiation times: (a, c) 400 s and (b, d) 800 s. Scale bars: 1 μm.

**Figure 4:**
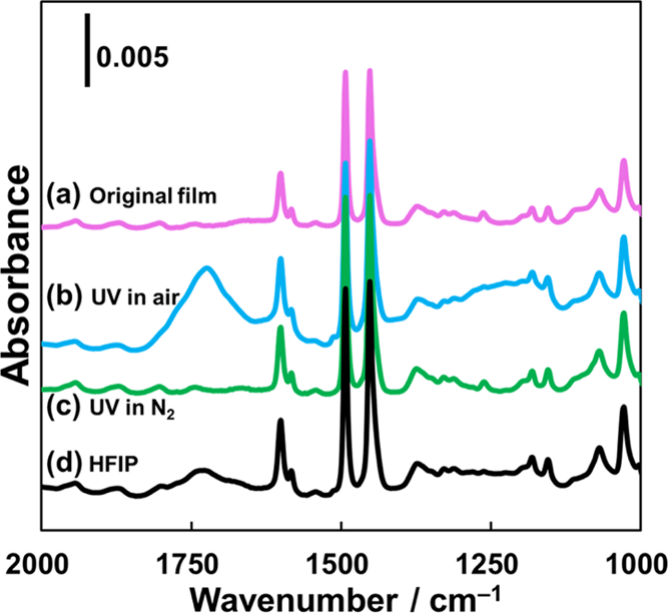
FTIR spectra of (a) the original PS film and PS films irradiated with UV light under (b) air and (c) N_2_, and (d) the UV-irradiated PS film after HFIP treatment.

We clearly demonstrated that illuminating the PMMA CPs/PS film with UV light effectively forms holes, where the UV light is focused by inducing polystyrene photodecomposition; however, how hole shape is influenced by HFIP is still unclear. In this study, HFIP, which is a good solvent for PMMA but a poor solvent for PS, was used to remove the PMMA CPs; in a parallel experiment, these CPs were also removed from UV-irradiated PS films with adhesive tape. Comparing the hole shapes formed by removing PMMA CPs with adhesive tape (Figure 3a,b) and HFIP (Figure 3c,d) revealed that UV irradiation alone produces holes in the PS films, with size apparently enlarged by HFIP treatment, which implied that the chemical composition of polystyrene surrounding the holes had changed to become HFIP-soluble; this change in composition was confirmed by FTIR spectroscopy of the PS films before and after UV irradiation. Figure 4 clearly shows that UV irradiation resulted in the new IR bands that are assigned to C=O and C–O stretching modes at ∼1740 and ∼1250 cm^–1^, respectively, which are associated with the photo-oxidized polystyrene moiety.^35,36,49−51^ The oxidization source is clearly aerobic oxygen since irradiation with UV light under N_2_ (Figure 4c) does not lead to hole formation. Furthermore, HFIP treatment (Figure 4d) led to fewer IR peaks that correspond to oxidized products due to the dissolution of the oxidized polystyrene regions surrounding the holes, resulting in hole enlargement. Interestingly, weak oxidation-related peaks were still observed even after HFIP treatment (Figure 4d), which indicated that some insoluble oxidized residues remained in the hole surroundings.

### 3.2 Hole Patterns Produced by Oblique Illumination

**Figure 5:**
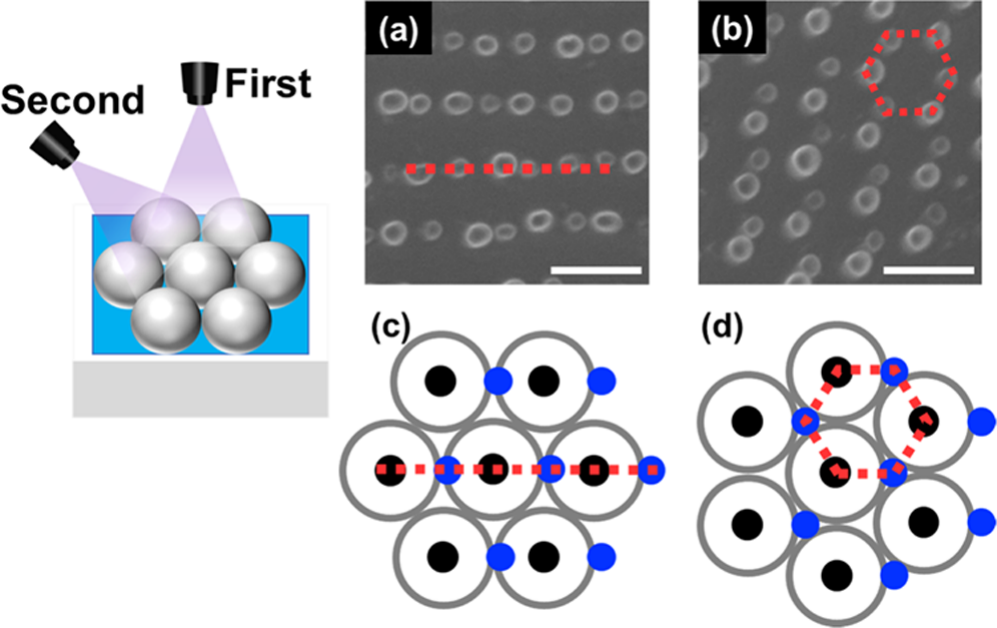
(a, b) Two holes carved by irradiating with UV light from two incident angles: 0 and 45°. (c, d) Schematic illustrating the relationship between the UV azimuth angle and the PMMA CP array hole pattern. Scale bars: 1 μm.

**Figure 6:**
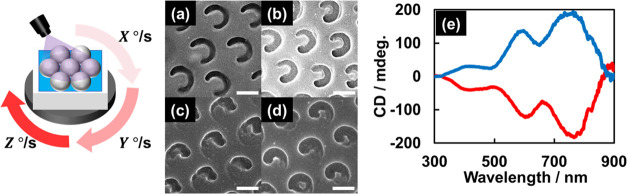
Schematic illustrating the preparation of nonspherical holes by irradiating UV light at an incident angle of 30° during PS-film rotation. (a–d) Effect of rotational speed over a total rotational angle of 270° on C-shaped and comma-shaped holes. UV intensity: 70 mW/cm^2^; rotational speed for each 90°: (a) 0.5, 0.5, and 0.5°/s, (b) 0.7, 0.7, and 0.7°/s, (c, d) 0.5, 1.0, and 2.0°/s. Rotational directions: (a–c) clockwise and (d) anticlockwise. (e) CD spectra of 30 nm Ag deposited comma-shaped holes prepared through clockwise and counterclockwise rotations. Scale bars: 1 μm.

Oblique UV irradiation enables various patterns to be drawn in conventional colloidal photolithography using photoresist films deposited on solid substrates.^26^ In the current system using PS films at air–water interfaces, oblique UV irradiation may form holes even in the vicinity of PMMA CPs by adjusting the azimuth and incident angles of the UV light. For example, two holes positioned directly beneath and to the side of a PMMA CP were generated from each particle by irradiating with UV light from two different incident angles: the normal and oblique directions (Figure 5). Linear-like (Figure 5a) and hexagonal-like (Figure 5b) arrays of holes were observed, with differences between the two types likely caused by the relationship between the UV azimuth angle and PMMA CP packing (Figure 5c,d). Furthermore, a curved hole pattern was carved by combining oblique irradiation with film rotation (Figure 6). Figure 6a shows C-shaped holes fabricated at an incident angle of 30°, a rotational speed of 1.5°/s, and a total rotational angle of 270°. The widths of the C-shaped holes were tunable by adjusting the rotational speed (Figures 6b and S5), as illumination power was easily controlled by adjusting the rotational speed of the sample film under constant-intensity UV light.

Tunability enables asymmetric chiral C-shaped holes to be carved (i.e., chiral comma-shaped holes) by changing the rotational speed every 90° rotation. The comma-shaped holes in Figure 6c were obtained using a 30° incident angle, 90 mW/cm^2^ UV light, three different rotational speeds (0.5, 1.0, and 2.0°/s) in sequence, and a total clockwise rotational angle of 270°. Because slower and faster rotational speeds (0.5 and 2.0°/s) resulted in wide and narrow lines, respectively, various comma shapes were carved using other sequences of rotational speeds (Figure S6).

Furthermore, the chirality of each comma-shaped hole was easily reversed by rotating counterclockwise (Figure 6d). In general, chiral nanostructures exhibit circular dichroism (CD); hence, films with comma-shaped holes were subjected to CD spectroscopy.^37−48^ However, only very weak CD responses were observed. Therefore, the chiral-hole films were coated with Ag thin films (30 nm) to enhance the CD signals because metallic chiral nano-objects usually exhibit strong CD signals.^52,53^Figure 6e shows that the CD signals were enhanced by Ag deposition, with the samples formed by clockwise and counterclockwise rotation showing opposite CD spectra.

### 3.3 Embossed Structures Prepared by Solvent Treatment

**Figure 7:**
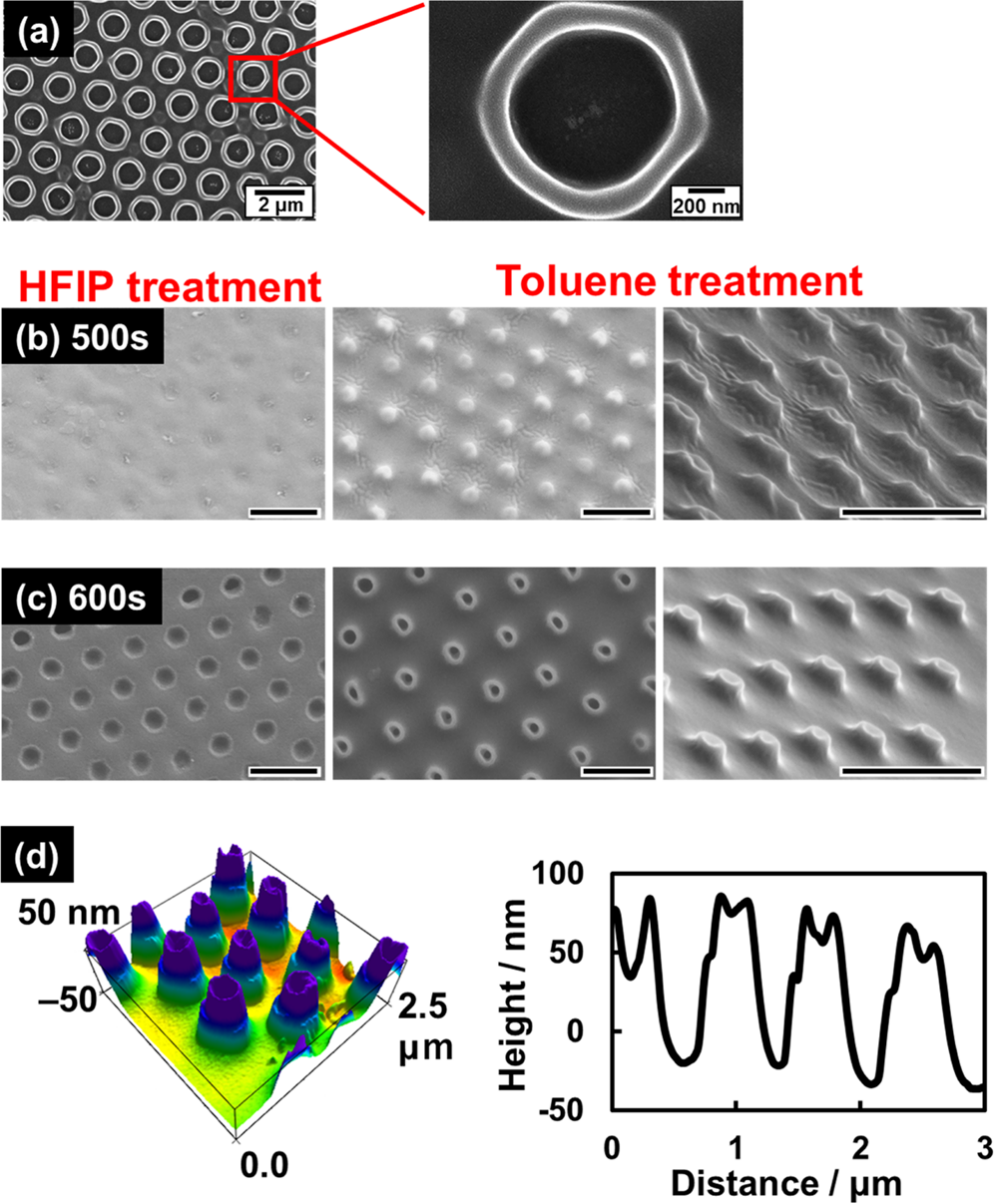
(a) SEM images of convex-ring structures formed by UV-irradiating PS films immersed successively in HFIP and toluene for 5 min. Intensity of UV light and exposure time: 70 mW/cm^2^ and 400 s, respectively. (b) PS films irradiated with weak UV light (10 mW/cm^2^) for (b) 500 s and (c) 600 s after immersion in HFIP for 5 min and then in toluene for 5 min. Scale bars: 1 μm. (d) AFM image and height profile of the UV-irradiated PS film after HFIP and toluene treatments: intensity: 10 mW/cm^2^; exposure time: 600 s.

We accidentally discovered that the rim of the photo-oxidized polystyrene residue swelled (Figure 7a) when the HFIP-treated hole-patterned PS film (Figure 2) was immersed in toluene for 5 min. The swelling of the HFIP-insoluble photo-oxidized residue was probably the result of solvent affinity that was distinguishable from that of the original nonoxidized PS, which enabled the morphology of the residue to be further modified by additional treatment with appropriate solvents.

**Figure 8:**
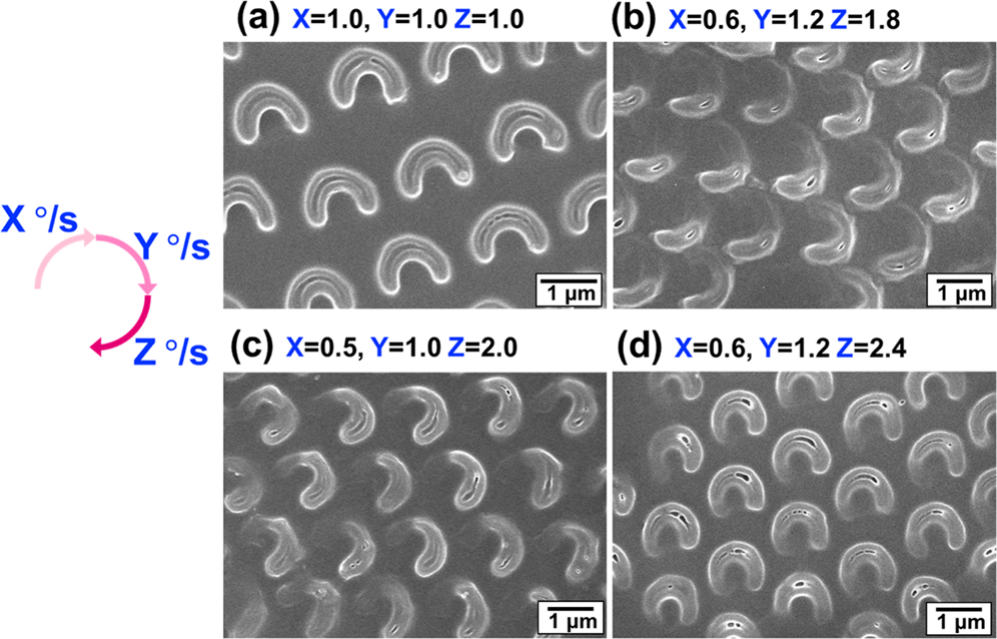
C-shaped and comma-shaped convex structures prepared by rotating PS films and immersing them in HFIP and toluene. UV incident angle: 30°, light intensity: 100 mW/cm^2^, total rotation: 270°. Rotational speed for each 90°: (a) 1.0, 1.0, and 1.0°/s, (b) 0.6, 1.2, and 1.8°/s, (c) 0.5, 1.0, and 2.0°/s, and (d) 0.6, 1.2, and 2.4°/s.

Weak PS photo-oxidation is the key to the observed swelling behavior because the considerably photodamaged domains produced by strong UV irradiation should be soluble in HFIP and eventually become holes. To produce spherical convex structures, PS films half-covered with PMMA CPs were exposed to weak UV light (10 mW/cm^2^) and then immersed in HFIP for 5 min and then in toluene for 5 min. Figure 7b–d shows that the toluene treatment yielded convex structures on the PS film, whereas the PS film prior to immersion in toluene exhibited shallow hollows. The heights of the convex portions depended on the UV exposure time; exposure times of 500 and 600 s led to heights of ∼30 and ∼100 nm, respectively. Accordingly, we successfully demonstrated that convex structures can easily be produced on PS films by tuning the UV exposure conditions followed by immersion in HFIP and toluene. Furthermore, C-shaped convex structures were also realized by oblique UV irradiation together with PS-film rotation; however, most of the centers of these convex shapes exhibited tearing (Figure 8). While improving the method for preparing C-shaped convex structures without tearing is a future challenge, we expect that this procedure will serve as a novel method for modifying the surfaces of polymer films.

### 3.4 Patterning Ag NPs Embedded in PS Films

**Figure 9:**
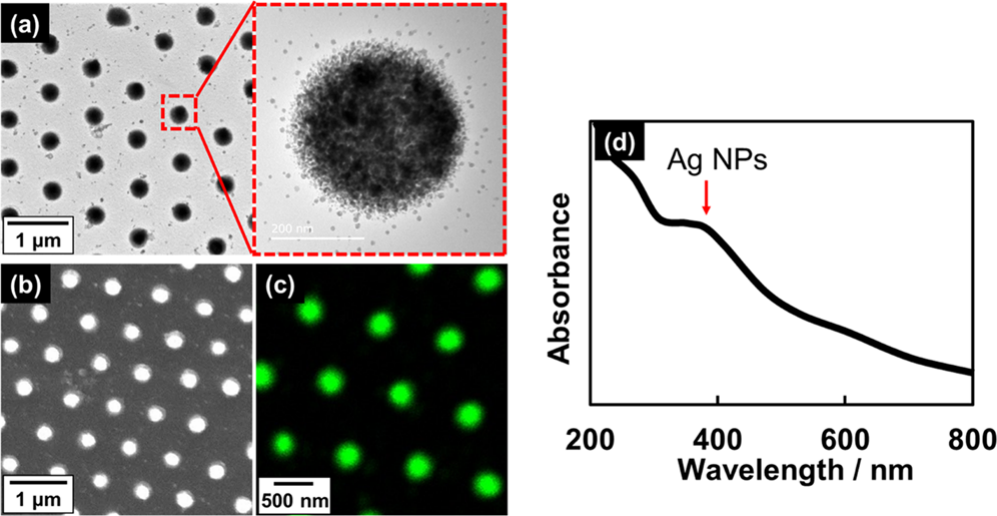
(a) TEM and (b) SEM images, (c) Ag TEM-EDS map, and (d) UV–vis spectrum of a PS film irradiated with UV light on a 10 mM aqueous solution of CH_3_COOAg.

The distinctive feature of the current method is that a water surface is used as a nanoprocessing field, in addition to the use of polystyrene instead of photoresists. The use of the water surface as the processing field in colloid photolithography enables photoreactive compounds to be deposited at UV-focused spots on PS films simply by dissolution in water. To embody this concept, we examined depositing Ag spots on PS films through the photoreduction of Ag ions dissolved in the water phase.^54,55^ PMMA CPs (800 nm) covering PS films floating on aqueous solutions of CH_3_COOAg (1 mM) were irradiated with UV light (50 mW/cm^2^) for 400 s. Figure 9a shows SEM, TEM, and scanning TEM-energy dispersive X-ray spectroscopy (STEM-EDS) elemental maps of UV-irradiated PS films after the PMMA CPs had been removed with HFIP. The electron microscopy images show that Ag ions in water produced periodic ∼300 nm diameter spots in 800 nm intervals, which corresponds to the natural diameter of a PMMA CP. Each spot is composed of NP aggregates (Figure 9a) that were confirmed to be metallic silver by TEM-EDS (Figure 9c) and XRD (Figure S7a). Furthermore, the formation of metallic Ag NPs was also confirmed by the appearance of a broad absorption peak at ∼410 nm in the UV–vis spectrum of the UV-irradiated PS film, assigned to the plasmonic Ag NP band (Figure 9d).^54,55^ Ag NPs, as photoreaction products, are deposited in specific domains directly under the PMMA CPs. Furthermore, a judicious choice of UV incident angle and PS-film rotation can be used to regulate the morphology of the Ag NP aggregates (Figure S7b).

Ag NPs have unique optical properties that depend on shape, size, and interparticle distance due to surface plasmon resonance, and chiral arrays of plasmonic Ag NPs give rise to strong chiroptical responses. Hence, the present approach is expected to be a convenient technique for imparting plasmonic properties to desired positions of PS films. Further, we expect that the present technique will be applied to other polymer films and can be extended to other metal and photoreactive compounds. Accordingly, using the water surface as the substrate provides a new technique for decorating polymer nanosheets with photoreactive compounds and may lead to considerable advances in colloidal photolithography.

## 4 Conclusions

PS films half-covered with PMMA CPs at the air–water interface were subjected to colloidal photolithography. Illumination with 250 nm UV light, which corresponds to the PS absorption band, resulted in photodamaged PS-film domains, with periodic spherical hole structures realized in the PS films by removing the photodamaged domains and PMMA colloids by HFIP washing. Regulating the illumination conditions by changing the UV incident angle and by rotating the PS film enabled nonspherical holes, including C-shaped and chiral comma-shaped holes, to be fabricated. We also showed that convex patterns can be formed by combining weak UV illumination with immersion in toluene. Furthermore, colloidal photolithography on the water surface is advantageous for producing Ag NP patterns on PS films by simply dissolving Ag ions in water. Accordingly, we clearly demonstrated that the use of PS films and water surfaces enables the further development of conventional colloidal photolithography.

## Supporting Information Available

SEM images of PS films covered with PMMA CPs before UV irradiation; SEM image of a hole pattern produced by UV irradiation of PS films covered with PMMA CPs on the glass substrate; photographic images of UV-irradiated PS films half-covered with PMMA CPs scooped from water onto various substrates; SEM images of UV-irradiated PS films at various UV light intensities; SEM images of UV-irradiated PS films at various total illumination energy densities; SEM images of C-shaped holes; SEM images of comma-shaped holes; and XRD pattern and TEM images of Ag-patterned PS films (PDF)
