# Supplementary material for: Hole, Convex, and Silver Nanoparticle Patterning on Polystyrene Nanosheets by Colloidal Photolithography at Air–Water Interfaces
Source: Langmuir. 2022 Jun 22;38(26):8153–9. doi: 10.1021/acs.langmuir.2c01069 (PMC9261183; doi:10.1021/acs.langmuir.2c01069)
Supplement: Supplementary file 1 — la2c01069_si_001.pdf [file la2c01069_si_001.pdf]

SUPPORTING INFORMATION

Hole, Convex, and Silver-Nanoparticle Patterning on Polystyrene Nanosheets by Colloidal Photolithography at Air–Water Interfaces

*Rino Kaneko, Hiroto Ichikawa, Marika Hosaka, Yoshihiro Sone, Yoshiro Imura, Ke-Hsuan Wang, Takeshi Kawai**

Department of Industrial Chemistry, Tokyo University of Science,
1-3 Kagurazaka, Shinjuku-ku, Tokyo 162-8601, Japan

Email: kawai@ci.tus.ac.jp

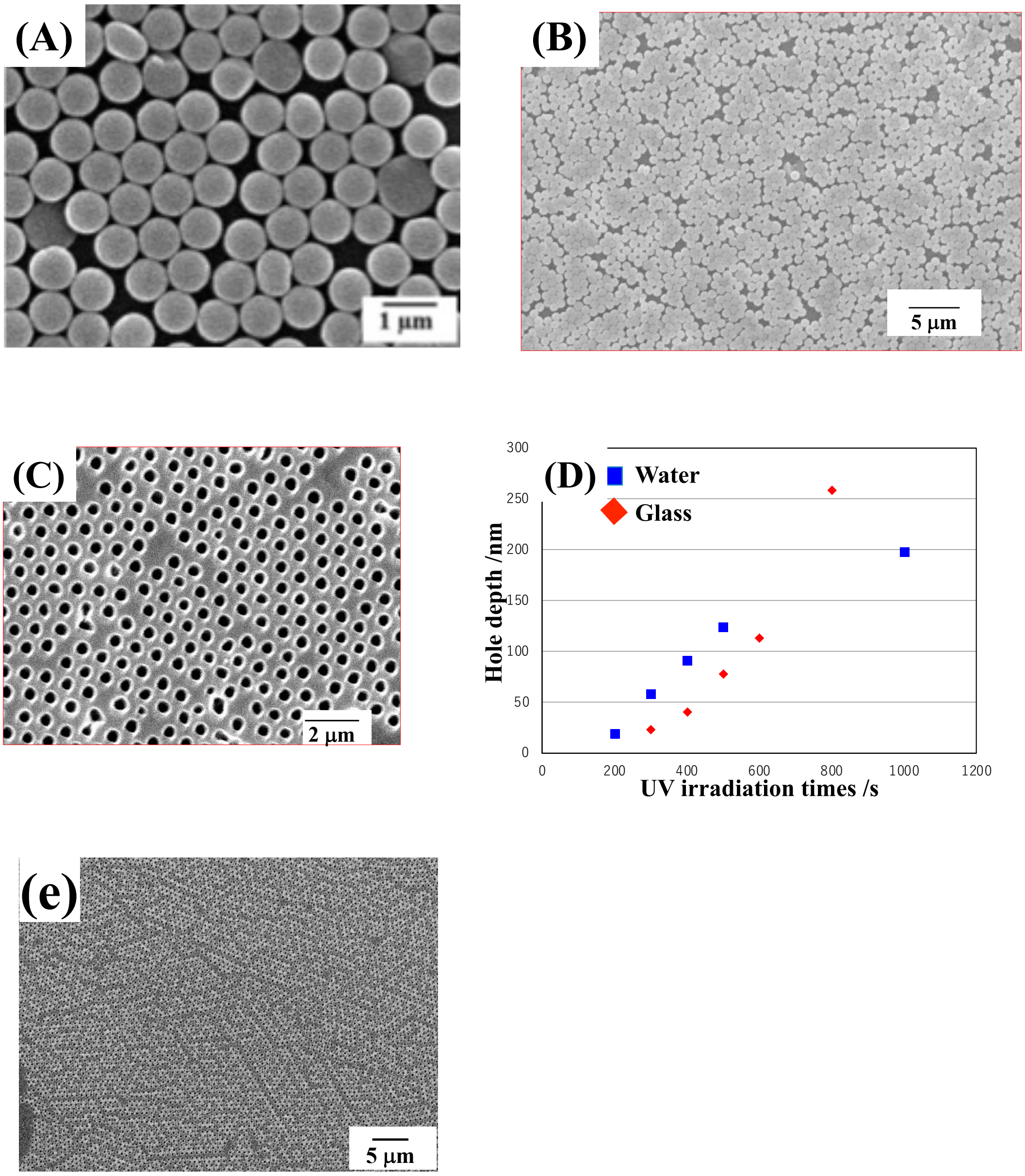

Figure S1. (a, b) SEM images of PS films covered with PMMA CPs before UV-irradiation. (c) SEM image of a hole pattern produced by UV irradiation of PS films covered with PMMA CPs on glass substrate after removing the PMMA CPs with HFIP. (d) Relationship between UV irradiation times and hole depth. (red) glass substrate and (blue) water surface. (e) SEM image of a large-area hole pattern produced by UV irradiation of PS films covered with PMMA CPs on water after removing the PMMA CPs with HFIP.

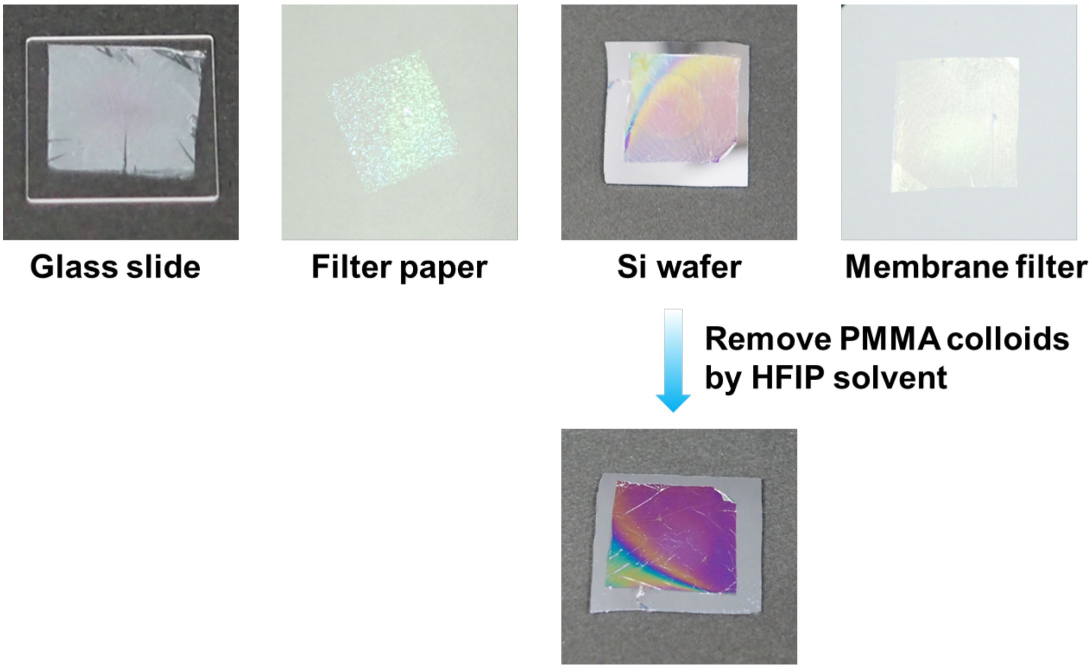

Figure S2. Photographic images of UV-irradiated PS films half-covered with PMMA CPs scooped from water onto a glass slide, filter paper, a silicon wafer, and a membrane filter.

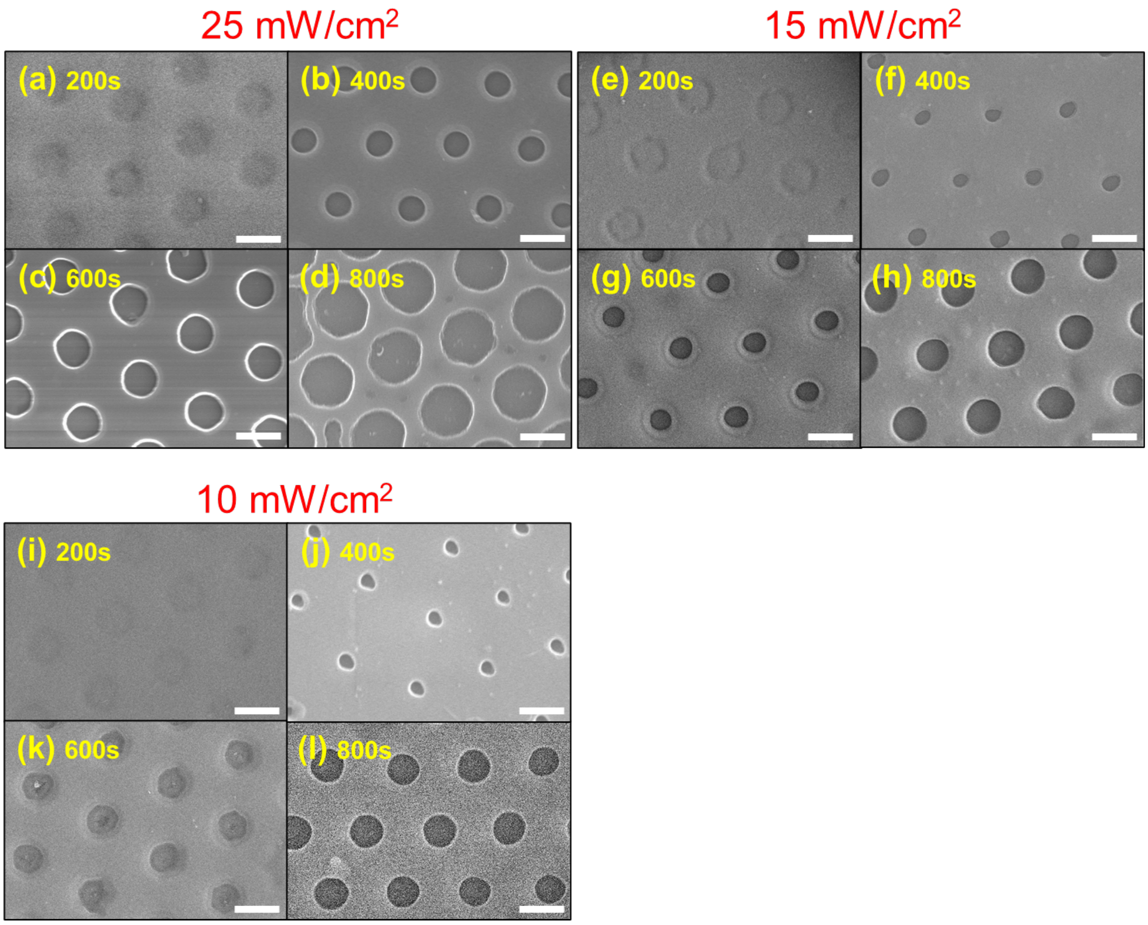

Figure S3. SEM images of UV-irradiated PS films after removing the PMMA CPs with HFIP. PMMA CP diameter: 1500 nm; PS film thickness: ~250 nm. UV light (250 nm) intensities: (a–d) 25 mW/cm^2, (e–h) 15 mW/cm^2, and (i–l) 10 mW/cm^2. Scale bars: 1 μm.

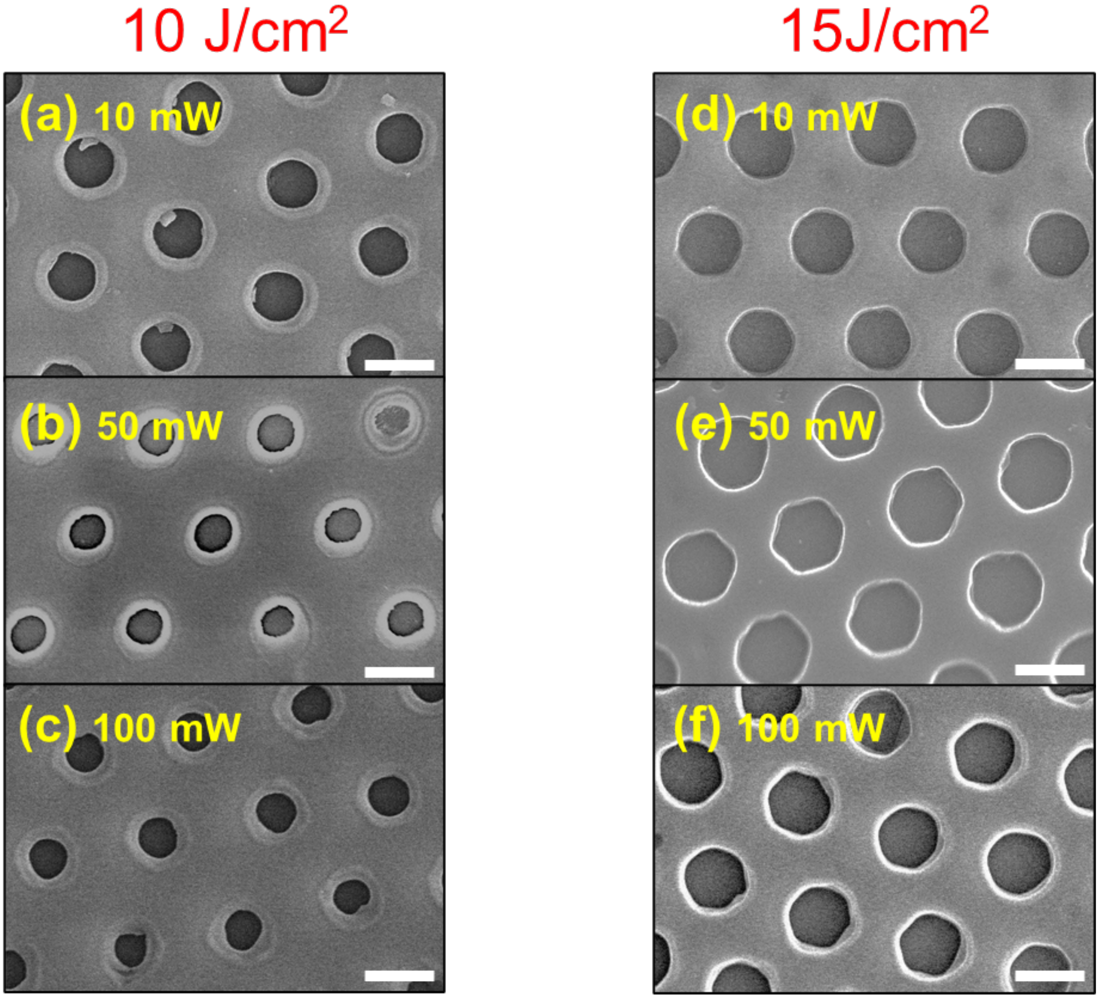

Figure S4. SEM images of UV-irradiated PS films after removing the PMMA CPs with HFIP. Total illumination energy densities: (a–c) 10 J/cm^2 and (d–f) 15 J/cm^2; (a) 10 mW/cm^2 for 1000 s, (b) 50 mW/cm^2 for 200 s, (c) 100 mW/cm^2 for 100 s, (d) 10 mW/cm^2 for 1500 s, (e) 50 mW/cm^2 for 300 s. and (f) 100 mW/cm^2 for 150s. Scale bars: 1 µm.

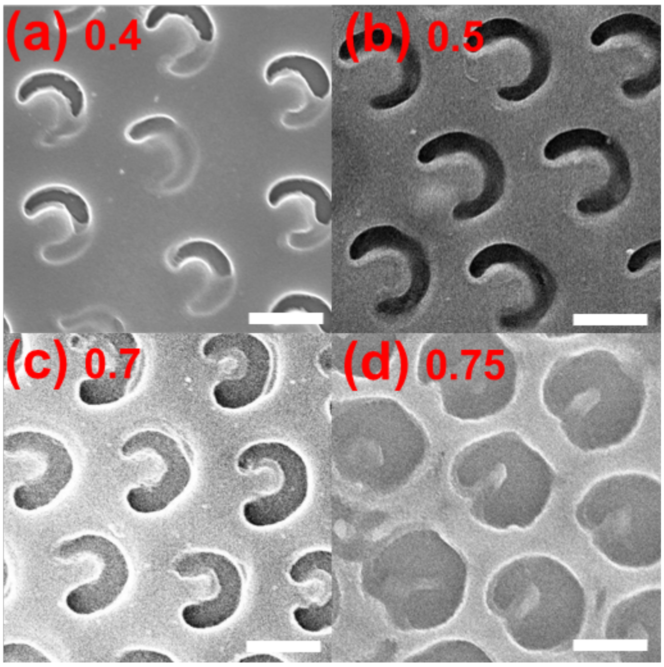

Figure S5. SEM images of C-shaped holes. Incident angle: 30°, UV light intensity: 70 mW/cm^2, total rotational angle: 270°, and rotational direction: clockwise. The rotational speeds: (a) 0.4 (b) 0.5, (c) 0.7, and (d) 0.75 °/s. Scale bars: 1 μm.

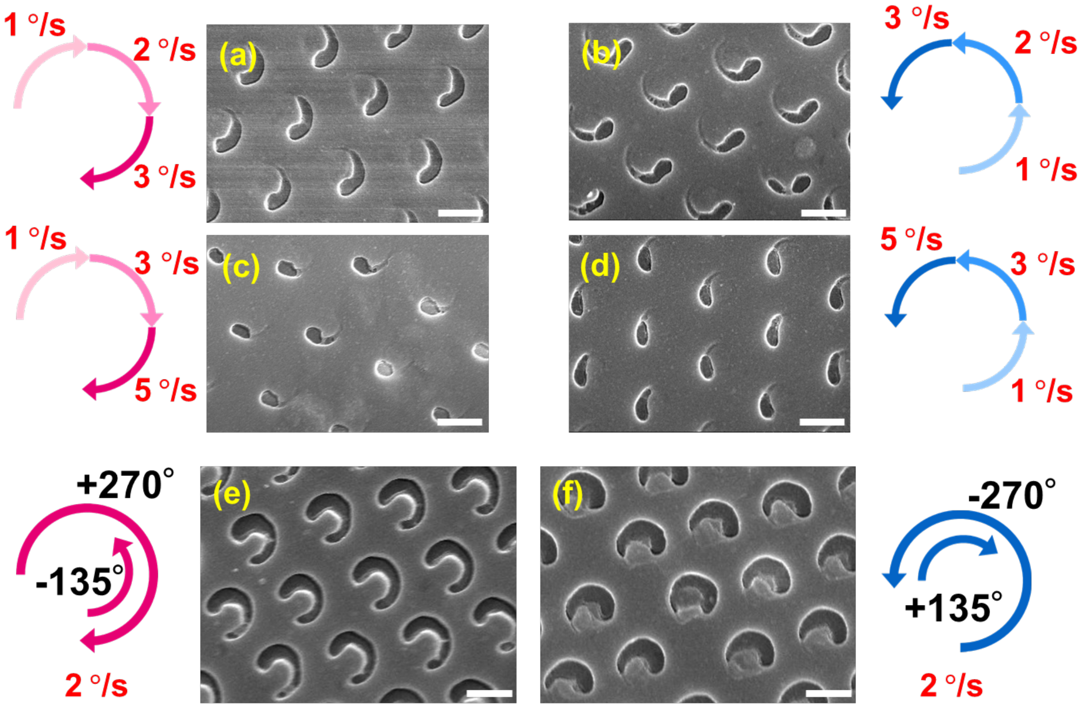

Figure S6. SEM images of comma-shaped holes. Incident angle: 30°, UV light intensity: 80 mW/cm^2, total rotational angle: 270°. Rotational directions: (a, c) clockwise and (b, d) counterclockwise. Rotational speed for each 90°: (a, b) 1.0, 2.0, and 3.0 °/s, and (c, d) 1.0, 3.0, and 5.0 °/s. (e) Clockwise rotation of 270° followed by counterclockwise rotation of 135° and (f) vice versa, at a constant rotational speed of 2.0 °/s. Scale bars: 1 μm.

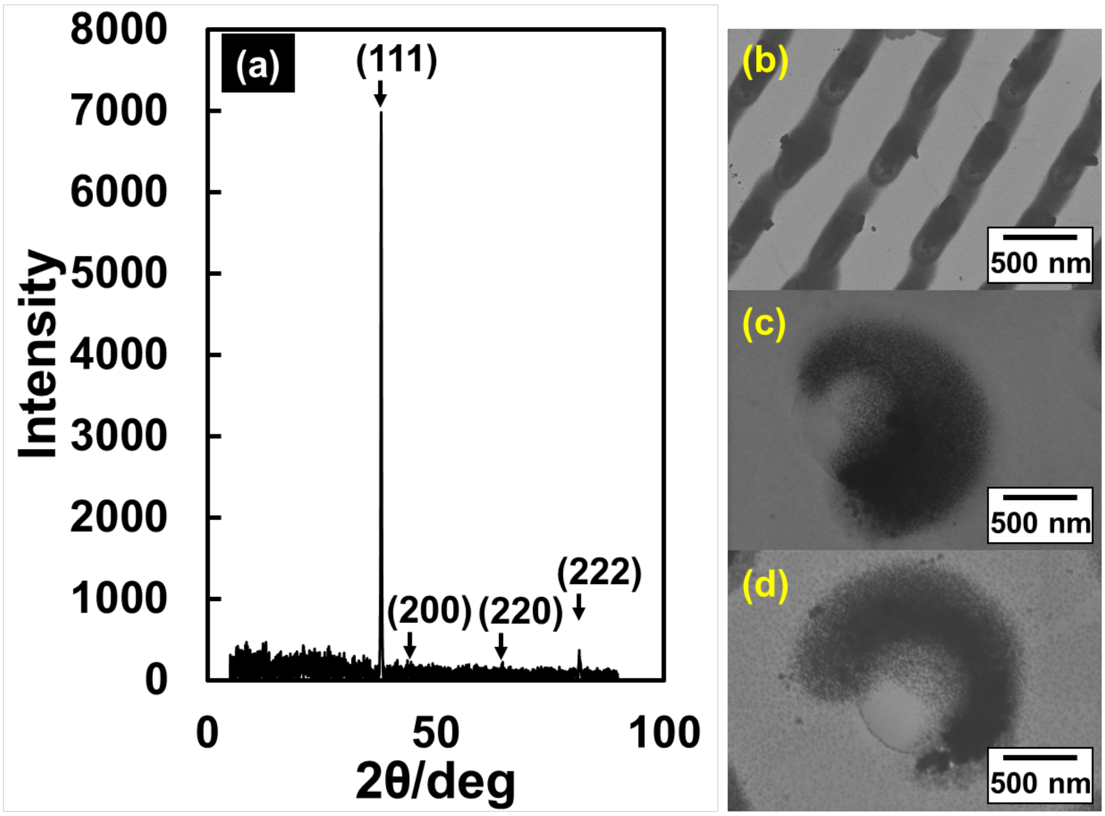

Figure S7. (a) XRD pattern of a PS film irradiated with UV light on a 10 mM aqueous solution of CH_3COOAg. (b–d) TEM images of Ag NP patterns on PS films. (b) PS film irradiated with UV light as the incident angle was changed from 45° to –45°. UV light intensity: 50 mW/cm^2, change in incident angle: 0.11 °/s. (c, d) Incident angle: 30°, total rotational angle: 270°, rotational direction: clockwise. UV light intensities and the rotational speeds: (c) 100 mW/cm^2 and 0.6 °/s and (d) 150 mW/cm^2 and 0.9 °/s. CH_3COOAg concentration: 1 mM.
